# Scoring Originality in Mathematical Problem-Solving: Comparison of Criterion-Referenced Scoring with Alternate Measures

**DOI:** 10.3390/bs16020249

**Published:** 2026-02-09

**Authors:** A. Kadir Bahar, Iclal Can, C. June Maker, Rabia Sipahi, Yasemin Sipahi

**Affiliations:** 1 Department of Educational Psychology, College of Education, University of Georgia, Athens, GA 30602, USA; yasemin.sipahi@uga.edu; 2 Guidance and Psychological Counseling Program, Middle East Technical University Northern Cyprus Campus, 99738 Güzelyurt, Türkiye; iclal@metu.edu.tr; 3 Disability & Psychoeducational Studies, College of Education, University of Arizona, Tucson, AZ 85721, USA; junemaker@hotmail.com; 4 Department of Finance, University of Illinois Chicago, Chicago, IL 60607, USA; rabias@uic.edu

**Keywords:** originality, mathematical creativity, creativity, scoring originality, teaching for creativity, creativity assessment

## Abstract

The purpose of this study was to examine the extent to which criterion-referenced originality scores are related to scores generated through alternative measures of originality (i.e., sample-based scoring and expert-referenced scoring) in mathematical problem-solving tasks. Drawing on data from 520 students enrolled in a public elementary school situated in a culturally diverse metropolitan area of New South Wales, Australia, the criterion-referenced approach was compared psychometrically with sample-based and expert-referenced scoring approaches. Another focus for analysis was on how each scoring system describes the relationship between originality and fluency. The results are important for ongoing debates about creativity and educational assessment, highlighting the implications of scoring methods for the interpretation of students’ original mathematical thinking. The study contributes important information for the design of fair and meaningful assessment and scoring practices.

## 1. Introduction

Originality has been recognized consistently as the most important component of creativity, an essential element of divergent thinking skills, and the most salient predictor of innovation (Acar et al., 2017; Guilford, 1982; Lubart et al., 2013; Runco & Acar, 2012). Scholarly interest in originality has remained central within creativity research since the mid-twentieth century (Guilford, 1950; E. P. Torrance, 1972; Wilson et al., 1953); however, due to increasing societal demands for innovation and novel solutions to major global problems, originality has begun to gain attention from researchers across disciplines. Yet even with its recognized importance, originality has been addressed insufficiently in educational practices and research, largely constrained by systematic barriers such as curriculum standardization, assessment accountability pressures, and lack of validated measurement tools (Bullard & Bahar, 2023).

Evidence from numerous studies shows that promoting school-aged children’s originality in mathematics education offers multiple benefits (C. J. Maker et al., 2023). Fostering originality helps advance the field itself and enables students to view mathematics as a dynamic, inquiry-driven discipline rather than a static set of rules (Devlin, 2000; A. K. Bahar et al., 2024). It also supports students’ holistic growth and nurtures their engagement with learning. By holistic growth, we refer to the development of students’ cognitive, affective, and dispositional capacities in mathematics, not just their procedural skills or content knowledge, but also their curiosity, confidence, flexible thinking, and willingness to engage with open-ended problems. Emphasizing originality encourages students to see mathematics as a space for exploration and meaning-making, thereby supporting broader intellectual and motivational development. In addition, emphasizing originality equips students with the skills to contribute to future discoveries and innovations, increases their positive attitudes toward creativity and original thinking, and helps them develop more accurate schema representations of creativity (C. J. Maker et al., 2023). Given these benefits, an important question is how originality in mathematical problem solving can be assessed most effectively. However, research on assessing and scoring mathematical originality remains limited (Leikin, 2013). This lack of clear assessment and scoring approaches often hinders its inclusion in the curriculum as well, which makes integration into everyday teaching and assessment challenging (Bullard & Bahar, 2023).

### 1.1. Originality in Mathematical Problem Solving

Creativity is commonly defined as producing ideas that are both novel and appropriate for the task (Runco & Jaeger, 2012). In school mathematics, appropriateness corresponds to mathematical validity and coherent reasoning. As an important dimension of creativity, we use originality to address the novelty component within mathematically valid responses, meaning solutions that go beyond routine, grade-level classroom methods while remaining mathematically sound. Literature suggests that when students construct and justify their own methods, rather than reproduce an algorithm, they engage in deeper reasoning and develop more flexible strategy knowledge that supports learning and transfer (Lithner, 2008; Rittle-Johnson & Star, 2007).

We also conceptualize mathematical problem solving as engaging with tasks for which the solution pathway is not immediately obvious and in which students must draw upon conceptual understanding and strategic decision-making. This perspective aligns with foundational formulations by Polya (1957) and later extensions by Schoenfeld (1985), who emphasize that problem solving involves interpreting the structure of a task, selecting or creating appropriate representations, and adapting strategies. Contemporary perspectives from the National Council of Teachers of Mathematics (NCTM, 2014) and the OECD (2019) similarly describe problem solving as a dynamic process requiring sense-making, exploration of relationships, and the formulation and testing of ideas. This broader theoretical grounding is important for interpreting originality within problem-solving contexts, as originality is not separate from problem solving but emerges through students’ strategic and representational choices.

Within this theoretical frame, originality in mathematical problem solving can be understood as the production of solutions that deviate from routine procedures by incorporating insight, representational diversity, or unconventional combinations of operations (Leikin, 2013; Silver, 1997). Originality reflects students’ ability to reorganize known concepts, reinterpret constraints, or identify patterns and analogies that enable new solution paths. Prior research shows that original ideas tend to emerge when students engage in deeper reasoning rather than simply enumerating possibilities (A. K. Bahar et al., 2024; Leikin, 2013). Thus, examining originality within problem-solving contexts requires attention to how students navigate task features, what kinds of insights tasks afford, and how the measurement framework recognizes conceptual rather than merely quantitative aspects of divergent thinking. School mathematics involves problems with standard answers and familiar solution procedures. In these settings, originality does not mean producing mathematics that is new to the discipline. Instead, it refers to novelty relative to routine strategies that are typically emphasized in the classroom. Research on creative mathematical reasoning suggests that students learn more deeply when they construct and justify a method rather than imitate an algorithm (Lithner, 2008). Work in mathematics education also frames novelty and flexibility as dispositions that can be fostered through problem solving and problem posing (Silver, 1997). Related evidence shows that working with multiple solution methods can support procedural flexibility and help students adapt strategies to new tasks (Rittle-Johnson & Star, 2007). For these reasons, we treat originality as a meaningful outcome variable even when problems have well-established solution families.

This empirical study has been rooted in the need for alternative measures for assessing originality that enable its consistent use with school-aged children. We have investigated the scoring of originality in mathematical problem-solving by comparing criterion-referenced scoring with alternative measures. Criterion-referenced scoring was selected as the primary point of comparison as it evaluates originality based on explicit task-defined criteria rather than normative responses or statistical rarity. In this study, criterion-referenced scoring is compared with sample-based scoring and expert-referenced scoring, which are regarded as two commonly employed approaches in the originality literature. 

A related question about assessing originality is its relationship with fluency, a topic that has been debated in the literature. In traditional divergent-thinking (DT) assessments, fluency and originality are often positively associated because scoring procedures typically link the number of ideas generated to the likelihood of producing statistically infrequent ones (e.g., Dumas & Dunbar, 2014; Forthmann et al., 2020; Mouchiroud & Lubart, 2001; Plucker et al., 2011). However, this structural dependency does not reliably apply to mathematical problem-solving tasks. Prior studies show that students often generate large sets of minimally varied solutions in open-ended numerical tasks, producing high fluency without corresponding increases in originality, while others generate only a few but substantially innovative responses (A. K. Bahar et al., 2024). Leikin’s work further demonstrates that high fluency does not guarantee creative or insightful mathematical output; students may produce many algorithmic or routine responses, whereas more original or insightful ideas often emerge from slower, deeper engagement with the task (Leikin, 2009). These domain-specific patterns create theoretical ambiguity regarding how fluency and originality should relate in mathematical contexts. Consequently, examining the empirical association between these two dimensions is essential for understanding the nature of mathematical originality and for evaluating whether different scoring approaches indicate fluency-dependent or fluency-independent aspects of creative problem solving. Accordingly, in this study, we also aim to explore whether the correlation between originality and fluency varies with different scoring methods.

### 1.2. Scoring Children’s Originality

Within the divergent thinking and creativity literature, originality is typically conceptualized as the uniqueness of ideas, and many foundational approaches operationalize this novelty through statistical infrequency (Alabbasi et al., 2022; Goff & Torrance, 2002; Guilford, 1950; E. P. Torrance, 1974; Silvia et al., 2008). Although appropriateness is a critical criterion in defining creativity as a broader construct, originality itself refers specifically to the novelty dimension of creative performance. At the same time, more recent scoring traditions recognize that novelty can be expressed not only through response rarity but also through insightfulness or conceptual sophistication (Leikin, 2013).

It is also important to note that uniqueness can be operationalized in several different ways within the literature. Some approaches classify responses in a binary manner (unique versus non-unique), whereas others employ multi-level scales that distinguish degrees of rarity based on the percentage of students who generated a given idea (Mouchiroud & Lubart, 2001; Silvia et al., 2008). Studies vary widely in the thresholds they adopt, with some characterizing responses as “rare” if they appear in fewer than 5 or 10 percent of cases, and others using broader cutoffs such as the 15–40 percent ranges proposed by Leikin (2013). These differences in operational definition can substantially influence the distribution and comparability of originality scores, as the same idea may be classified differently depending on the reference group and rarity criterion (Plucker et al., 2011). Such methodological variation further underscores the importance of examining alternative scoring approaches that do not depend exclusively on frequency-based definitions of uniqueness.

Three main scoring techniques are commonly used to assess children’s originality: sample-based, norm-referenced, and expert-referenced. These methods are presented next, along with their strengths and limitations. In addition, in this empirical paper, we introduce criterion-referenced scoring as an alternative measure by presenting its potential to address some of the limitations of other approaches.

#### 1.2.1. Criterion-Referenced Scoring

The first and primary method for scoring originality examined in this study is criterion-referenced scoring, which we propose as an alternative approach to evaluating originality. Unlike norm-referenced scoring, in which students’ work is compared with that of others, in criterion-referenced, pre-determined standards are used (Neil et al., 1999) to evaluate originality. These criteria may be developed by teachers and aligned with curriculum standards, rather than using statistically based norm groups as in a norm-referenced process or expert judgments as in an expert-referenced process.

The use of criterion-referenced scoring enables the assessment of originality in everyday student work, and, importantly, connects standards-based teaching with teaching for creativity, two areas often seen as conflicting. However, criterion-referenced scoring has both advantages and disadvantages; one drawback is that considerable time and effort are needed to develop and apply procedures (Neil et al., 1999).

#### 1.2.2. Sample-Based and Norm-Referenced Scoring

Sample-based scoring is another method used to assess originality. In sample-based scoring, originality is defined relative to the specific cohort of students completing the task, meaning that a response is considered original when it is uncommon or unique within that sample (K. Bahar & Maker, 2025; Mouchiroud & Lubart, 2001). The sample can be any group, such as a single classroom, a mathematics enrichment program, or an intervention cohort responding to a common mathematical task.

Like other scoring methods, sample-based scoring also has limitations, particularly related to the nature of the sample, such as its diversity and size, which can restrict the range of original responses within the group (Plucker et al., 2011). For example, in homogeneous or small groups, an idea may appear original when only a few students attempted varied strategies. Similarly, in more advanced groups, highly original ideas may be under-recognized if multiple individuals produce similar uncommon solutions.

Norm-referenced scoring may represent a specific form of sample-based scoring, in which originality is evaluated using a large normative sample. It is one of the methods most frequently used in creativity research to evaluate originality. One of the most widely used applications of norm-referenced scoring is found in the Torrance Tests of Creative Thinking (TTCT; E. P. Torrance, 1974, 1990, 2008; T. F. Torrance, 1998), which are the divergent thinking tests used most often. Originality in the TTCT is scored by counting ideas that are statistically infrequent. The use of normative data shows how often each response occurs (Alabbasi et al., 2022; E. P. Torrance, 1974, 1990, 2008; T. F. Torrance, 1998). When a response is not on the normative list, it is considered original and assigned a point (Alabbasi et al., 2022).

Despite its potential for tracing patterns in creative thinking as well as offering a systematic method for assessing originality, norm-referenced scoring has limitations too. One limitation is that normative data used for scoring can lose relevance over time and cannot fit different age groups very well (Mouchiroud & Lubart, 2001). These normative data can also reflect culture-specific response patterns, making generalization across cultural contexts difficult (Alabbasi et al., 2022). In addition, a norm-based system can miss the creative value of an idea that is statistically common but still innovative and meaningful in a particular classroom. On the other hand, some responses may appear original because they are uncommon; however, they may not be appropriate for the given task. Thus, statistical rarity alone may not be sufficient to indicate originality. For these reasons, considering temporal, developmental, and cultural differences, as well as contextual appropriateness, is important when using norm-referenced scores to identify originality. Furthermore, given that norm-referenced scores typically are not provided to teachers for either summative or formative use, they remain largely impractical for routine classroom application.

#### 1.2.3. Expert-Referenced Scoring

Another common approach to assessing originality is expert-referenced scoring (Silvia et al., 2008). In this method, experts with domain-specific expertise evaluate responses for their originality (Baer, 2017). Unlike norm- or sample-referenced methods, expert-referenced scoring is accomplished by experts who evaluate the originality of ideas within a given context. It is widely regarded as one of the gold standards for assessing originality, particularly in complex or domain-specific contexts in which such judgment is essential. One well-known example of this approach is the Consensual Assessment Technique (CAT; Amabile, 1982, 1996), in which domain experts evaluate the originality of an outcome.

Expert-referenced scoring has its own limitations, particularly the resources required to recruit and coordinate qualified experts, and the potential for subjectivity stemming from individual biases or differing interpretations of creativity. Moreover, implementing scoring procedures among experts requires a lot of time, effort, and manual work (Silvia et al., 2008). Furthermore, this method might not be very convenient in typical classroom contexts because finding experts for routine summative and formative assessments is an impractical task for teachers.

We also want to note that, although the CAT was originally conceptualized as a method in which domain experts evaluate the creativity or originality of products (Amabile, 1982, 1996), subsequent research has noted that non-expert raters can also provide reliable evaluations, particularly when sufficient training or anchoring examples are provided (Silvia et al., 2008; Plucker et al., 2011). The literature shows that expertise enhances the depth and sensitivity of judgments, but expert status is not an absolute requirement for producing consistent ratings under CAT procedures (Amabile, 1996). In the present study, we employed domain experts because the task involved mathematical reasoning, and we aimed to ensure that judgments of originality highlighted the conceptual quality and sophistication of students’ ideas.

Overall, the literature presented here highlights both the importance of originality in mathematical problem solving and the ongoing challenges associated with its scoring. Although sample-based and expert-based approaches offer valuable insights into the assessment of originality, the assumptions underlying these methods (e.g., expert judgments, sample dependence) may limit their use in the classroom. An important question that requires in-depth exploration is whether originality can be meaningfully assessed through criterion-referenced scoring, which may play a crucial role in strengthening the measurement of originality in mathematical problem solving and contributing to its systematic integration into mathematics classrooms.

### 1.3. Rationale and Significance of the Present Study

Assessing originality in mathematical problem-solving has long posed conceptual and methodological challenges in creativity research and education. Although originality is considered a fundamental element of creativity (Guilford, 1982; Lubart et al., 2013), how it is made operational and measured varies across studies and contexts (A. K. Bahar et al., 2024). Use of traditional scoring systems, including sample-based approaches, tends to be based on the idea of originality as a statistical rarity and to give higher scores to infrequent responses (Mouchiroud & Lubart, 2001). While such methods have demonstrated utility in research contexts, they frequently combine productivity and creativity, relying on comparative samples that may not reflect meaningful or contextually appropriate originality (Mouchiroud & Lubart, 2001). In this context, we have introduced and provided evidence for the validity of a criterion-referenced approach to originality scoring, thus measuring conceptual quality and relevance of ideas according to predefined criteria rather than sample-specific frequencies.

One of the significant aspects of this study is its contribution to construct validation. By empirically examining the degree of convergence between criterion-referenced and alternative methods, the authors explore whether criterion-referenced scoring reflects comparable or different dimensions of original thinking. If originality scores obtained via criterion-referenced and other scoring indices have strong alignment, this finding would support the argument that criterion-referenced procedures can approximate other methods while having greater transparency and applicability for classroom practice. On the other hand, if correlations are moderate or weak, criterion-referenced scoring may be a measurement of a qualitatively different dimension of originality. In either case, the validation effort provides research-based psychometric evidence to a field in which measurement practices often are based on tradition rather than empirical examination.

This study is also important for creativity and divergent thinking research because we address an enduring theme in the literature: the fluency-originality relationship. Decades of empirical work have shown that measures of idea fluency (the number of ideas generated) correlate strongly with originality scores when scoring is sample-based (Mouchiroud & Lubart, 2001). Such relationships, however, have resulted in concerns about discriminant validity, as high fluency can increase originality scores even when ideas lack conceptual depth or novelty. By comparing the fluency-originality correlation using different scoring methods, the authors contribute to the understanding of whether criterion-referenced scoring better differentiates originality from fluency. If criterion-referenced scoring yields weaker or non-significant correlations with fluency, relative to sample-based scoring, this would indicate that criterion-referenced scoring reduces the statistical artifact of originality. Thus, it may be a more selective and theoretically coherent method for assessing original thinking.

Beyond its psychometric implications, this study has practical significance for mathematics education. Despite increasing emphasis on fostering creativity in children, originality in mathematical problem-solving often has been neglected in K-12 curricula due to several systematic barriers (Bullard & Bahar, 2023), including a lack of appropriate and convenient methods of assessment available to teachers (A. K. Bahar & Maker, 2020; K. Bahar & Maker, 2025). Teachers and curriculum developers frequently struggle to assess creativity within problem-solving tasks because norm-referenced, sample-based, and expert-referenced scoring approaches require normative data, comparison groups, or panels of experts that are not always available to everyday classroom teachers (Mouchiroud & Lubart, 2001). In contrast, use of a standards-based criterion-referenced framework allows teachers to evaluate students’ original mathematical ideas against grade-level expectations and task-specific criteria by aligning creativity assessment with curriculum standards while reducing dependence on external samples. By embedding criterion-referenced originality scoring into mathematical problem-solving tasks, and by extension into other STEM-related problem-solving contexts, educators can systematically identify students’ creative strengths and provide targeted feedback. They can cultivate classroom cultures in which multiple solutions, ways of solving problems, and original answers are valued (Devlin, 2000; C. J. Maker & Pease, 2021).

Finally, the study has the potential to provide data for broader assessment reform and policy initiatives. In an era in which 21st-century skills are emphasized, educators seek reliable ways to document creative problem-solving without compromising validity or comparability (C. J. Maker et al., 2021). Criterion-referenced scoring can help meet this goal by providing a usable and theoretically grounded approach to assessing originality (K. Bahar & Maker, 2025). The evidence generated from this study can be a guide for educators and policymakers about assessment design, professional development for teachers, and large-scale evaluation of creative learning outcomes.

### 1.4. Purpose and Research Questions

In this study, we aim to explore the extent to which criterion-referenced originality scores are related to scores generated through alternative measures of originality (i.e., sample-based scoring and expert-referenced scoring) in mathematical problem-solving tasks. Given the debates about originality and fluency in the literature on children’s originality, we also aim to explore whether the correlation between these two constructs varies across different scoring methods. The following research questions guided this study:

1. To what extent are originality scores derived from criterion-referenced scoring correlated with alternative methods in mathematical problem-solving tasks?
2. How does the relationship between fluency and originality in mathematical problem-solving vary when evaluated using different originality scoring methods?

## 2. Method

### 2.1. Research Setting

The data used in this study were collected during the 2015–2016 academic year as part of a project in which the Real Engagement in Active Problem Solving (REAPS) model was implemented in a multicultural public elementary school in New South Wales, Australia. The project was approved by the New South Wales State Education Research Application Process (SERAP; 2013149) and supported by the University of Arizona Institutional Review Board. REAPS is a combination of four evidence-based approaches: Problem-Based Learning (PBL), Discovering Intellectual Strengths and Capabilities while Observing Varied Ethnic Responses (DISCOVER), Thinking Actively in a Social Context (TASC), and the Prism of Learning, each designed to foster problem-solving skills (C. J. Maker & Pease, 2021). Although initially created for gifted and talented learners, the REAPS model has been shown to help educators promote creative problem-solving in all students (e.g., Alhusaini, 2016; A. K. Bahar et al., 2021; Gomez-Arizaga et al., 2016; J. Maker & Bahar, 2024; C. J. Maker et al., 2021, 2022; Wu et al., 2021). Details on the implementation and research on the REAPS model are provided in C. J. Maker and Pease (2021) and C. J. Maker and Zimmerman (2025).

### 2.2. Participants

In this study, we analyzed data from 520 students enrolled at the participating elementary school. The sample included 122 second graders, 124 third graders, 101 fourth graders, 93 fifth graders, and 80 sixth graders. Students were included if their mathematics assessment scores were available and if both parental consent and student assent had been obtained. The school served a student body characterized by considerable ethnic, linguistic, and socioeconomic diversity; however, individual demographic data could not be collected due to restrictions of the research protocol.

### 2.3. Instruments

Originality in mathematical problem solving was scored using the DISCOVER mathematics assessment instrument (C. J. Maker, 2005; Tan & Maker, 2020), which was administered in a paper-and-pencil format. The assessment tool was designed to align with students’ academic developmental levels and was available in grade-specific forms (Pre-K, K–2, 3–5, 6–8, and 9–12). In this study, only three forms, the K–2, 3–5, and 6–8, were used. The K–2 version included 14 tasks (6 closed, 7 semi-open, and 1 open), whereas the 3–5 and 6–8 versions each contained 17 tasks (9 closed, 7 semi-open, and 1 open). Although the DISCOVER assessment provides three grade-level versions of the open, semi-open, and closed mathematical problems, the structure and purpose of the tasks remain constant across grades. In all versions, closed tasks involve grade-appropriate operations and a magic square, semi-open tasks require students to use a given set of numbers to generate as many correct problems as possible, and open tasks invite students to write multiple mathematically valid problems that produce a specified answer. Only the numerical values and difficulty levels vary developmentally. For example, the open problem requires students to generate problems equaling 10 in the K–2 version, 18 in the Grades 3–5 version, and 24 in the Grades 6–8 version. Despite these surface differences, the underlying generative structure and creativity affordances are equivalent across grade levels, which supports analyzing the open-ended responses together and interpreting fluency, flexibility, and originality within a common conceptual framework. Detailed descriptions of the task types and their theoretical underpinnings are included in prior studies (A. K. Bahar & Maker, 2011; A. Bahar & Maker, 2015; A. K. Bahar et al., 2021; Griffiths, 1996; Sak & Maker, 2005; Sarouphim, 1999; Tan & Maker, 2020).

**Figure 1:**
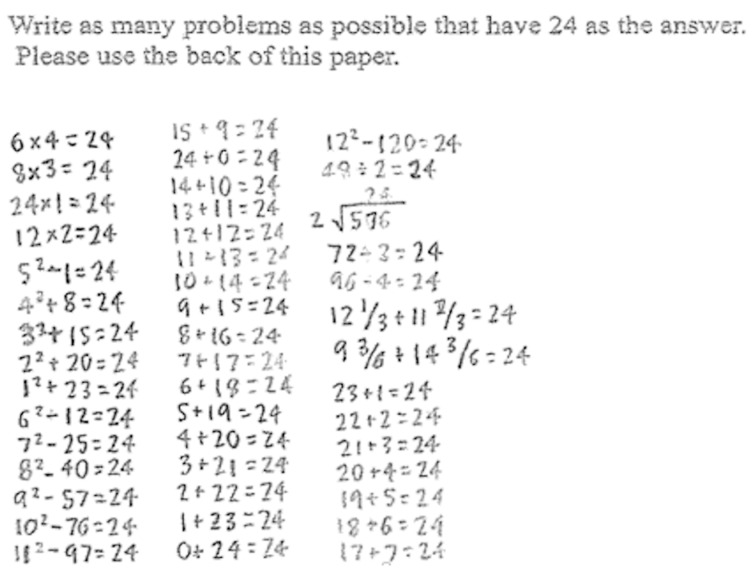
Example of an open problem with a 6th-grade student’s solution. From “What does it take to be original? An exploration of mathematical problem-solving” (A. K. Bahar et al., 2024). Reproduced with permission from Elsevier, with license number 6037631317699.

Aligned with the purpose of our study, we evaluated only student solutions to the open problems (See Figure 1 for an example of open problems). The open-ended task was not timed, and students were permitted to work at their own pace. Observational records indicated that most students invested substantial time in this task. On average, students spent approximately 10–15 min generating solutions, although some worked for considerably longer, with individual times extending up to one hour. This open time structure allowed students to develop solutions without the constraints of time pressure.

Evidence from prior research has provided support for the assessment’s psychometric soundness: for example, Sak and Maker (2005) found that DISCOVER scores collected at the beginning of the academic year accounted for 20% of the variance in Stanford Achievement Test, Ninth Edition (Stanford-9) mathematics scores at the end of the year, demonstrating predictive validity. Moreover, inter-rater reliability has consistently been high, with coefficients reported at 0.94 (Griffiths, 1996; C. J. Maker, 2005; Sarouphim, 1999).

The open-ended task shown in Figure 1 represents one example of a mathematical problem-solving task designed to elicit multiple solution strategies. Although this task reflects only one form of problem solving, it was selected because it enables students to demonstrate representational originality qualities (Silver, 1997; Leikin, 2009). Open-ended tasks of this type have been widely used in creative problem-solving research because they allow students opportunities to construct solutions rather than apply predetermined algorithms.

### 2.4. Data Scoring

#### 2.4.1. Scoring Originality

In this study, we scored student originality in mathematical problem solving via three different methods: sample-based, expert-referenced, and criterion-referenced scoring.

*Sample-based scoring.* Sample-based originality scoring followed the framework developed by Leikin (2009), which evaluates the rarity of a student’s overall response set relative to the distribution of solutions generated by the entire sample. Similar to Leiken’s methodology, solution types were classified based on their frequency within the sample: rare (<5%), uncommon (5 to 15%), moderately common (15 to 40%), and common (>40%). These thresholds are not fixed population criteria; rather, they are sample-dependent classifications consistent with the logic of sample-based scoring in prior creativity research (Reiter-Palmon et al., 2019; Said-Metwaly et al., 2021).

To make this scoring operational, we applied a system similar to Leikin’s decimal-based scale. Solutions that were rare (<5%) received a score of 10; solutions that were uncommon (~5 to 15%) received 3 points; solutions that were moderately common (~15 to 40%) received 1 point; and, finally, a common solution (>40%) received 0.1 point. A student’s overall originality score was calculated as the sum of the originality values assigned to all their solutions for a given task. For example, a student who produced 1 rare solution, 2 uncommon solutions, 5 moderately common solutions, and 34 common solutions will receive a total score of 24.4 points.

Two expert researchers, both former mathematics teachers, conducted the sample-based scoring. All student responses were first compiled and grouped into distinct solution types. The frequency of each solution type across the full sample was calculated, and each type was assigned to one of four rarity categories (rare, uncommon, moderately common, or common). Raters worked collaboratively and reached consensus on each classification. Finally, a total originality score was obtained for each student by adding all the separate scores that were assigned for each solution.

*Expert-referenced scoring*. In addition to sample-based scoring, we scored originality in students’ mathematical solutions using an expert-referenced procedure grounded in the Consensual Assessment Technique (CAT; Amabile, 1982). In this approach, domain experts assess originality, not by predetermined statistical rarity thresholds but through holistic comparisons across the set of student solutions. They use their professional expertise and judgement in the scoring process. Such expert-referenced scoring is recognized widely as a valid method in creativity research because its basis is informed consensus of individuals with expertise in the domain (Baer & McKool, 2009; Barth & Stadtmann, 2021). Using CAT, expert judges view student artifacts produced in response to open-ended tasks. They rate each artifact on how original (or novel) they believe it is, in comparison to other artifacts in the same set. Usually, no absolute benchmark is provided; the rating is relative to the group of products being evaluated.

Expert-referenced originality ratings followed standard Consensual Assessment Technique (CAT) procedures and were standardized in three ways. The expert panel consisted of three raters, including two educational researchers and one mathematics teacher with more than 15 years of teaching experience. First, each rater worked independently, scoring student work without consulting the other raters to preserve the independence of judgments. Second, all raters received the full set of student solutions for each form, allowing them to compare each student’s work to that of all other students. Third, the order of student work was randomized for each rater to avoid order effects. Raters examined all of a student’s solutions and assigned a single holistic originality score reflecting the originality of the most original solution in the student’s set. They used an eight-point scale ranging from 1 (very uncreative) to 7 (very creative), consistent with CAT practice. Judges were given unlimited time to complete the task to ensure thoughtful and deliberate evaluations. The final expert-referenced originality score for each student was the mean of the three raters’ independent holistic ratings.

To evaluate whether the expert-referenced scores could be treated as a reliable aggregate measure, we calculated intraclass correlation coefficients (ICC) based on the three raters. A two-way random-effects model was used. The reliability of a single rater was ICC(2,1) = 0.62, and the reliability of the mean rating across the three expert raters was ICC(2,3) = 0.81, 95% CI [0.75, 0.88], indicating that the aggregated expert-referenced score was a dependable composite. Just a side note for the readers, the term “single-rater reliability” refers to the dependability of one rater’s score relative to the other rater under the ICC(2,1) model; it does not imply that reliability was assessed for only one rater, but rather that reliability was calculated for individual ratings instead of the mean of both raters.

The raters for the expert-referenced scoring and the criterion-referenced scoring were not the same individuals. Expert-referenced scoring was completed by highly experienced mathematics educators trained in the use of the Consensual Assessment Technique, whereas criterion-referenced scoring was conducted by a separate set of trained evaluators who applied the predefined originality criteria. Keeping rater groups separate prevented potential cross-contamination between scoring frameworks.

Because the expert-referenced method required judges to view each student’s complete set of solutions, we acknowledge that weaker responses within a set may be visible during scoring. However, raters were specifically instructed to base their judgment on the highest level of originality demonstrated in the set, rather than on the overall average quality of responses. This approach aligns with standard CAT practice, in which raters form a holistic impression of a student’s most creative contribution (e.g., Silvia et al., 2008). Although each participant receives only one score from each rater, the ICC reflects the degree to which raters consistently differentiate among students, i.e., produce similar rank-orderings of originality across participants, which is why reliable estimates can be obtained even when ratings are holistic and single-valued.

*Criterion-referenced scoring*. As the final method, we scored each student’s originality based on a standard set of criteria without reference to other students. In this method, an original solution is defined as production of a response that deviates from common solution methods prompted by instructor/teacher/standards in the classroom, considering the grade level content and standards prior to the scoring process. For example, in the open problem (Figure 1), students were asked to write as many problems as possible that have 24 as the answer. For this problem, a solution that included only basic facts and operations (e.g., 1 + 23 = ?, 25 − 1 = ?) was not original, whereas an uncommon solution, such as the use of fractions, decimals, roman numerals, symbolic representations, algebraic notations, and story, was scored as original for a student in first grade.

The criteria for our task were developed by two experts, one experienced math teacher and one researcher. The criterion-referenced rubric evaluated the overall originality demonstrated in each student’s entire set of solutions across four dimensions of mathematical novelty, which were created by the team. These dimensions can be conceptualized as how originality can be manifested in solutions:

(a) novel use of operations (e.g., multiplication, division, or unexpected combinations of operations),
(b) novel use of numbers (e.g., use of fractions, decimals, or non-standard number systems such as Roman numerals),
(c) novel use of representations (e.g., symbolic, algebraic, geometric, or narrative representations), and
(d) novel use of reasoning (e.g., inductive or deductive reasoning, analogical reasoning, or systematic trial-and-error).

Each dimension was scored on a 0–5 scale based on the highest level of novelty observed across the student’s full set of solutions. A score of 0 indicated that the student’s solutions relied exclusively on common, routine, or teacher-modeled strategies. A score of 1 indicated evidence of one type of novelty within that dimension, 2 indicated two types of novelty, 3 indicated three types, 4 indicated four types, and 5 indicated the presence of five or more novel features within that dimension. Importantly, these scores do not correspond to points assigned to individual solutions. Rather, each criterion reflects the breadth and depth of novelty shown across the student’s entire response set. The student’s criterion-referenced originality score was computed by summing the four-dimension scores, yielding a possible range of 0–20.

Before the actual scoring began, two raters scored 50 selected solutions. Later, the raters discussed discrepancies between their scores for each solution until they reached consensus on scoring procedures. Interrater reliability for each criterion was evaluated using a two-way random-effects intraclass correlation coefficient for agreement (ICC[2,1]), appropriate for continuous ratings. Agreement was strong across all four criteria: ICC = 0.86 for novel use of operations, ICC = 0.80 for novel use of numbers, ICC = 0.93 for representations, and ICC = 0.79 for reasoning. After the raters made certain their scoring process was consistent, each of them scored all student solutions. Later, a final originality score for each student was obtained by averaging each rater’s originality score. Interrater reliability for the composite criterion-referenced originality score of a single rater was ICC(2,1) = 0.78, and the reliability of the mean of the two raters was ICC(2,2) = 0.88, indicating that the aggregated criterion-referenced score is highly reliable.

#### 2.4.2. Scoring Fluency

As is common in creativity research, our operational definition of fluency was the ability to produce many different correct solutions to a mathematical problem. We counted the number of correct solutions that a student produced for a given problem. The average number of correct solutions for the given open problem was slightly higher than 23 (Table 1). Students received one fluency point for each correct solution. Prior to reaching consensus, the inter-rater reliability coefficient was 0.94 for fluency scores.

### 2.5. Data Analysis

To address the research questions, quantitative analyses were conducted. The dataset was screened for missing values before analysis. Because fewer than 1% of data points (three cases) were missing, these were excluded from further analyses (Field, 2005). Descriptive statistics and correlation matrices were first computed to provide an overview of the data distribution and initial associations among the scoring methods (See Table 1 and Table 2).

For the first research question, pairwise Pearson correlation coefficients were calculated to determine the extent to which originality scores obtained from the three scoring procedures were related. Differences in correlation strength were examined to determine the degree of convergence or divergence across scoring methods.

For the second research question, correlational and comparative analyses were conducted to explore how the relationship between fluency and originality varied depending on the scoring approach used. Specifically, separate correlations between fluency and originality were computed within each scoring method. To evaluate whether these relationships differed significantly across methods, Fisher’s *r*-to-*z* transformations were applied. Effect sizes were also reported to assist in interpreting the magnitude of differences.

As a side note, several approaches in the divergent thinking literature propose adjusting originality scores for fluency by calculating an average originality index. However, such corrections are most appropriate for additive rarity-based originality scores and do not generalize well to rater-based or criterion-referenced approaches, which evaluate conceptual quality independently of response quantity (Silvia et al., 2008; Forthmann et al., 2020). In the present study, the sample-based method also minimizes the influence of fluency because Leikin’s scoring system assigns values based on the insightfulness and unconventionality of each solution rather than simply tallying the number of rare responses. As a result, producing more responses does not automatically lead to higher sample-based originality scores unless those responses reflect conceptually meaningful novelty. Because the three scoring frameworks differ in their theoretical foundations and do not share a common additive structure, applying a fluency correction would reduce conceptual comparability and distort the interpretive meaning of two of the three methods. For this reason, we examined the relationship between fluency and originality directly rather than applying a correction.

## 3. Results

The descriptive statistics for fluency and the three originality scoring methods are presented in Table 1. Substantial variability occurred across participants, with fluency scores showing the largest dispersion (M = 23.32, SD = 15.37). Among the originality measures, sample-based originality displayed the greatest range, whereas expert-referenced and criterion-referenced originality scores were lower and less variable. Overall, these findings show meaningful differences among participants in both the fluency and originality of their ideas, providing a foundation for exploring how fluency relates to originality across scoring methods.

Pairwise Pearson correlation coefficients were computed to examine how closely these are measures of the same underlying construct of originality in mathematical problem solving. The results in Table 2 show that all three measures were highly and positively correlated (*p* < 0.001), suggesting strong relationships across scoring approaches. The highest correlation was observed between expert-referenced and criterion-referenced (*r* = 0.89), indicating that expert judgments align closely with the structured novelty criteria used in criterion-referenced scoring. The sample-based measure also correlated strongly with both expert-referenced (*r* = 0.81) and criterion-referenced (*r* = 0.83), showing that sample-based originality scoring is consistent with expert and criterion-referenced evaluations. The corresponding effect sizes (*r*^2^ = 0.66–0.79) indicate substantial shared variance among methods.

In Table 3, correlations between fluency and originality are shown across the three scoring methods. Separate Pearson correlations were computed for each fluency and originality pairing, and Fisher’s *r*-to-*z* transformations were used to compare the relative strength of the associations. The relationship between fluency and originality varied significantly by scoring method. The sample-based originality had a moderate positive correlation with fluency (*r* = 0.45, *p* < 0.001). The criterion-referenced originality showed a weaker but still significant positive correlation (*r* = 0.15, *p* < 0.001), while the expert-referenced originality exhibited the smallest relationship with fluency (*r* = 0.09, *p* = 0.040).

Fisher’s *z* comparisons (z-difference tests) confirmed that the fluency and sample-based correlation was significantly stronger than both fluency and expert-referenced (z = 6.30, Δ*z* = 0.40, *p* < 0.001) and fluency with criterion-referenced correlations (z = 5.33, Δ*z* = 0.34, *p* < 0.001). The correlations between fluency and expert-referenced originality and between fluency and criterion-based originality did not differ significantly (z = 0.98). The corresponding effect sizes (*r*^2^ = 0.20, 0.01, 0.02) indicate that fluency explained substantial variance in sample-based originality scores but minimal variance in expert- or criterion-referenced originality.

## 4. Discussion

The purpose of this study was to examine the extent to which criterion-referenced originality scores are related to scores generated through alternative measures of originality (i.e., sample-based scoring and expert-referenced scoring) in mathematical problem-solving tasks. Across all analyses, the strong correlations among scoring methods (*r* = 0.81–0.89) suggest that each shares an underlying construct of originality, albeit through distinct interpretive lenses. This significant relationship indicates that originality, as a cognitive-creative attribute, can be recognized consistently whether defined by structured criteria, expert judgment, or statistical rarity. Such coherence across frameworks supports the construct validity of originality as a measurable outcome in students’ mathematical thinking, extending earlier arguments that multiple scoring traditions may converge when tasks elicit authentic creative reasoning (Mouchiroud & Lubart, 2001).

Perhaps one of the most important results of our study was empirical support for the validity of criterion-referenced scoring as a measure of originality in mathematical problem solving. The strong correlation with expert evaluations demonstrates that criterion-referenced scoring is a combination of two important constructs: originality and appropriateness. Moreover, its weaker association with fluency provides evidence for discriminant validity: criterion-referenced scoring is a way to distinguish between mere productivity and genuinely original thought (A. K. Bahar et al., 2024; Leikin, 2013). Thus, criterion-referenced scoring offers a balanced alternative, structured enough for reliability yet flexible enough to reflect the authentic nature of creative reasoning (K. Bahar & Maker, 2025). By aligning scoring criteria with curricular goals, criterion-referenced scoring allows originality to be both measurable and teachable, a critical step toward embedding creativity into everyday educational practice.

Overall, consistent with expectations, the three scoring approaches were intercorrelated at a high level, with the strongest relationship between expert-referenced and criterion-referenced scoring (*r* = 0.89). The importance of this result is that it shows that experts’ qualitative judgments of novelty are closely related to the structured, rubric-based criteria embedded in criterion-referenced. In both approaches, conceptual depth and appropriateness are given priority over statistical rarity, suggesting that criterion-referenced scoring can approximate expert consensus without the logistical burden of convening multiple raters. Relatedly, empirical support is provided for the argument that criterion-referenced scoring can serve as a viable, classroom-ready counterpart to expert evaluation (K. Bahar & Maker, 2025). Whereas expert-referenced methods are time-intensive and context-bound, criterion-referenced scoring offers scalability and transparency, allowing teachers to apply consistent standards of originality within the curriculum. Sample-based scoring, although strongly correlated with both expert-referenced and criterion-referenced approaches (*r* = 0.80), reflects a different emphasis. Its moderate association with fluency (*r* = 0.45) links productivity to originality, which is partly a statistical function of the number of ideas produced.

Many researchers have criticized sample-based scoring because they often result in relationships between fluency and originality that lead to an inability to separate two important constructs (Meier et al., 2021; Mouchiroud & Lubart, 2001). By contrast, the weak correlations between fluency and both expert-referenced scores (*r* = 0.09) and criterion-referenced scores (*r* = 0.15) show that these frameworks reflect the originality of solutions rather than their quantity. All three methods evaluated in this study (expert-referenced, criterion-referenced, and sample-based approaches) are measures of aspects of originality, but have an emphasis on different dimensions, divergent output versus conceptual novelty, underscoring the multidimensional nature of mathematical creativity.

Another central finding is the way each scoring method relates to fluency. Sample-based originality exhibited a moderate positive association with fluency (*r* = 0.45), whereas the links for expert-referenced and criterion-referenced scoring were weak (*r* = 0.09 and *r* = 0.15, respectively) and significantly smaller than the sample-based association (Fisher’s r-to-z differences ≈ 0.30–0.40, *p* < 0.01). Practically, when originality is defined by statistical rarity within a cohort, students who generate more responses tend to accumulate more original ideas; when originality is defined by expert judgment or by criteria, output volume is far less consequential. These contrasts replicate and extend concerns that additive, frequency-based originality indices are confounded with fluency, whereas approaches guided by expert judgments and criteria better isolate quality from quantity (Forthmann, 2024; Mouchiroud & Lubart, 2001; Osborn, 1953).

Researchers who rely on additive scoring of “rare” responses often report strong fluency to originality correlations and warn about poor discriminant validity; use of non-additive or central tendency approaches typically diminishes those correlations (Forthmann et al., 2020; Meier et al., 2021; Silvia et al., 2008). Our findings are aligned with that logic, with sample-based as a rarity-weighted additive index and expert-referenced or criterion-referenced approaches being more like qualitative filters that dampen the fluency signal (Mouchiroud & Lubart, 2001). Methodologically, then, the choice of scoring framework is not only a cosmetic preference: it also may shift the balance between “more ideas” and “novel ideas”. Having said that, although the sample-based method uses a rarity-weighted system that reduces the degree to which fluency drives originality scores, any summative scoring method inherently retains some fluency dependence. For example, two students who produce only rare solutions will differ in total score simply because one generated more responses. This structural dependency helps explain why the sample-based score correlated more strongly with fluency than the criterion-referenced or expert-referenced scores, which were non-summative and designed to evaluate the quality or level of the most original idea rather than the total number of ideas. Consequently, differences in correlation magnitude across scoring methods should be interpreted in light of these methodological differences. These findings are also consistent with prior research showing that fluency and originality are often less tightly coupled in mathematical problem-solving tasks than in traditional divergent-thinking tasks (A. K. Bahar et al., 2024; Leikin, 2009, 2013).

### 4.1. Implications for Creativity Research and Education

For researchers, we emphasize the need to make explicit the theoretical perspective guiding the way originality is scored. The significant relationship among scoring approaches is an indicator of a shared underlying construct, yet the differing relationships with fluency show how operational choices determine what counts as original. We recommend that future researchers examine how criterion-referenced scoring changes over time and across disciplines, and test whether criterion-referenced originality scores are useful predictors of meaningful creative achievement beyond the immediate task. Future studies could explore whether these scores are associated with students’ performance on unfamiliar or novel problems, correspond with teachers’ own judgments of originality, or remain stable in different contexts, tasks, and instructional processes. These research questions highlight the importance of longitudinal and classroom-based studies that can help clarify the dynamic relationships among originality scoring, instructional processes, and meaningful learning.

The strong correlation between expert-referenced and criterion-referenced scores offers encouraging evidence for the validity of the criterion-referenced approach; however, these findings should be interpreted with care. In this study, the scoring procedures were applied by individuals with substantial expertise in mathematics education, and such expertise likely contributed to the consistency and quality of scoring. Their success in developing and using the criteria does not automatically imply that teachers in typical classroom settings, who differ widely in experience, training, and familiarity with creative problem solving, would apply the criteria with the same reliability. Additional research is needed to determine the kinds of professional development, scaffolding, or implementation supports that would enable broader teacher use of criterion-referenced originality scoring in everyday practice. Thus, while promising, the approach should be viewed as a direction for future development rather than an immediately generalizable classroom tool.

Criterion-referenced scoring is also a pragmatic bridge between curriculum standards and originality. Clear originality criteria, co-developed and shared with students, can be guides for lesson design, supports for targeted feedback, and methods for cultivating a visible culture of original mathematical thinking across grade levels. Because criterion-referenced scoring does not depend on the rarity of responses within a particular cohort, students are not penalized when they are in classrooms rich in creative ideas or have the advantage of specific experiences.

Although the present study focused on open numerical problem-generation tasks, an important direction for future work is to examine how originality manifests across different mathematical domains. Not all areas of mathematics afford the same opportunities for creative variation. Domains grounded in procedural or discrete structures, such as geometric measurement (e.g., area, volume) or combinatorics, may constrain the range of possible original responses compared to domains with greater combinatorial flexibility, such as number operations or algebraic thinking. Prior research suggests that originality in mathematics is highly task-dependent and may be shaped by the representational demands of the content area (A. Bahar & Maker, 2015; Leikin, 2009). Therefore, future studies should investigate whether the three scoring approaches examined here function similarly across domains, or whether certain domains elicit different fluency–originality relationships or different distributions of rare versus common solution types. Such work would deepen our understanding of how mathematical creativity develops across content areas and could inform domain-specific instructional design.

A growing body of research suggests that the creativity observed in students’ mathematical responses is fundamentally shaped by the structure and cognitive demands of the tasks they are given. According to the Cognitive Task Framework (A. Bahar & Maker, 2015; Stein & Smith, 1998), tasks differ in the extent to which they require memorization, procedural application, or genuine problem solving. Tasks that are closed, highly scaffolded, or limited to a single correct method provide very few opportunities for creative variation. In contrast, open-ended tasks with multiple entry points and high-level cognitive demand, such as tasks that allow students to generate, represent, or transform ideas, tend to elicit greater originality. This perspective aligns with research on mathematical creativity, showing that novelty and flexible thinking depend heavily on the affordances built into the task itself (Leikin, 2009). Accordingly, the scoring methods examined in this study may function differently depending on whether tasks encourage combinatorial exploration (as in number operations) or rely on fixed procedures (as in standard measurement tasks). Future work should therefore attend carefully not only to scoring methods but also to how task design either enables or restricts creative expression.

Finally, an additional consideration concerns the untimed nature of the open-ended task. Because students could work for as long as they wished, fluency scores likely reflect not only divergent-thinking ability but also students’ motivation, persistence, and interest in the task. Research on divergent thinking shows that fluency is not a pure indicator of ability but is influenced by individual differences in engagement and willingness to generate many responses (Runco & Acar, 2012). Similarly, studies using open-ended creativity tasks suggest that task interest and personality factors can affect the number of responses produced (Silvia et al., 2008). Accordingly, fluency in the present study should be interpreted as a joint outcome of cognitive potential and motivational or situational engagement, which may partly explain the variability observed across participants.

### 4.2. Limitations

We urge readers to consider several limitations. First, although the strong relationship between expert-referenced scoring and criterion-referenced scoring methods provides evidence to strengthen the case for evaluation of originality based on clear criteria, correlation alone does not establish predictive validity. In future research, we recommend studies of outcomes such as students’ ability to solve novel problems, teachers’ in-class judgments of creative reasoning, and longitudinal indicators of creativity development. These studies can help to determine whether originality based on criterion-referenced scoring methods are predictors of meaningful learning and performance. Second, evidence from our study is derived from a mathematical problem-solving assessment. Other disciplines may have different relationships between originality and fluency. Extending criterion-referenced scoring to open-ended scientific modeling, engineering design tasks, or multi-step proof writing would be tests of its sensitivity to domain-specific markers of originality. These types of studies would also clarify how criteria can be adapted so that criteria and/or rubrics provide information about students’ novel insight rather than their use of familiar forms of sophistication within each field.

Third, the present study focuses on a single open-ended numerical task, which reflects only one form of mathematical problem solving. Other types of problem-solving contexts (e.g., modeling, multi-step reasoning, proof, geometric investigation) may evoke different patterns of originality. Future research should examine whether the relationships among scoring methods observed here hold across different problem-solving tasks, including problems requiring modeling, spatial reasoning, or proof-based justification.

A further limitation concerns the interaction between task instructions, students’ strategic responses, and the measurement of originality. Because the assessment prompted students to “write as many problems as possible,” the task naturally emphasized fluency, and fluency-oriented instructions are known to elicit rapid production strategies that often yield simple variations rather than deeper, insight-based responses (Forthmann et al., 2020; Silvia et al., 2008). This dynamic reflects what divergent thinking researchers refer to as instruction–scoring alignment: the validity of originality scores can depend on whether students were encouraged to focus on generating many ideas, generating original ideas, or both (Acar et al., 2020; Reiter-Palmon et al., 2019; Said-Metwaly et al., 2020). Studies on explicit instructional framing remain mixed; some report no differences under originality instructions (Acar et al., 2020), while others find that explicit originality prompts increase originality scores without altering fluency or flexibility (Said-Metwaly et al., 2020). In the present study, the fluency-oriented instructions likely encouraged speed-based strategies for some students, which may have influenced the distribution of originality scores. Because the assessment was part of a pre-existing standardized instrument, we did not have autonomy to modify or supplement these instructions to balance fluency and originality. Although our expert-referenced and criterion-referenced scoring methods mitigate this concern by evaluating conceptual novelty independently of response quantity (Amabile, 1996; Leikin, 2013), the trade-off between speed and originality cannot be fully separated from the task structure. Future research would benefit from comparing tasks with different instructional emphases (e.g., “generate as many original solutions as possible”) to determine how instructional cues shape strategic problem-solving behavior and the relationships among fluency, flexibility, and originality.

Finally, the authors did not include a norm-referenced approach, another common form of scoring originality. This was a deliberate design choice driven by the absence of a sufficiently large and representative normative pool. Because norm-referenced indices are dependent on sample size, age bands, and culture, a small pool can distort estimates of novelty and inflate or depress originality scores. Incorporating norm-referenced scoring along with expert-referenced and criterion-referenced approaches using multi-site, stratified, and periodically updated norms will be important additions to the literature about scoring originality.

## 5. Conclusions

In sum, relationships between the three originality frameworks were highly significant, with the alignment between expert-referenced scoring and criterion-referenced scoring especially strong, while the relationship between originality and fluency diverged meaningfully across methods. These patterns provide clarification for longstanding debates about originality scoring—scores based on relative originality tend to increase with quantity, whereas in expert-referenced or criterion-referenced approaches, what makes an idea novel is independent of the number of ideas produced.

Originality scoring can be a valid, scalable, and instructionally coherent way to evaluate creativity in everyday mathematics, empirically supporting the use of criterion-referenced scoring in school settings (K. Bahar & Maker, 2025). By embedding clear originality scoring criteria into routine assessments, creative mathematical thinking becomes visible and teachable, without waiting for expert panels or depending on shifting cohort norms. That shift may keep creativity within reach of classroom teachers while respecting the development of originality as an important goal of education.

## Figures and Tables

**Table 1:** Descriptive Statistics.

Scoring Method	N	M	SD	Min	Max
Fluency	520	23.32	15.37	0	111
Sample-based Originality	520	3.45	4.29	0	41.3
Expert-referenced Originality	520	1.88	1.50	0	7
Criterion-referenced Originality	520	3.09	2.91	0	16

*Note.* N = sample size; M = mean; SD = standard deviation.

**Table 2:** Correlations among Originality Scoring Methods.

Scoring Method	Sample-Based Originality	Expert-Referenced Originality	Criterion-Referenced Originality
Sample-based Originality	—	0.66	0.69
Expert-referenced Originality	0.81	—	0.79
Criterion-referenced Originality	0.83	0.89	—

*Note.* Below diagonal: Pearson’s *r*. Above diagonal: effect size *r*^2^. All correlations are significant at *p* < 0.001.

**Table 3:** Fluency—Originality Correlations, Fisher’s z Transformations, and z-Difference Comparisons.

Scoring Method	*r*	Fisher’s *z*	*r* ^2^	Fisher’s *z* vs. SB Δ*z*	*p* Value
Sample-based Originality	0.448	0.482	0.20	—	<0.001
Expert-referenced Originality	0.090	0.090	0.01	0.37	0.040
Criterion-referenced Originality	0.150	0.151	0.02	0.29	<0.001

*Note.* Fisher’s z = ½ ln ((1 + r)/(1 − r)). Δz values represent Fisher’s z differences compared with the fluency and sample-based originality correlation. All values computed using N = 520. Differences for sample-based originality and expert-referenced originality are significant at *p* < 0.001; Differences for sample-based originality and criterion-referenced originality are significant at *p* < 0.001 too; Differences for expert-referenced originality and criterion-referenced originality are nonsignificant.

## Data Availability

The data presented in this study are available on request from the corresponding author.
